# Endoscopic cadaveric analysis of the origin of the ophthalmic artery

**DOI:** 10.1007/s00276-023-03234-4

**Published:** 2023-08-18

**Authors:** Chunhui Zhou, Ting Lei, Junzhao Sun, Hulin Zhao, Xin Yu, Weidong Cao, Wenying Lv, Jianning Zhang

**Affiliations:** 1 Department of Neurosurgery, Sixth Medical Center of PLA General Hospital, Beijing, People’s Republic of China; 2 Department of Neurosurgery, Sanbo Brain Hospital, Capital Medical University, Beijing, People’s Republic of China

**Keywords:** Endoscopic endonasal approach, Optic nerve, Internal carotid artery, Ophthalmic artery, Distal dural ring

## Abstract

**Purpose:**

The ophthalmic artery is often involved in suprasellar and parasellar surgeries, but the anatomical structure where the ophthalmic artery originates has not been fully clarified from the perspective of an endoscopic endonasal approach (EEA).

**Methods:**

A total of 10 fresh cadaveric heads (20 sides) were dissected through an EEA, and the origin of the bilateral ophthalmic arteries and their adjacent structures were observed from a ventral view. The origin of the ophthalmic artery in 50 healthy people was retrospectively studied on computed tomography angiography imaging.

**Results:**

The ophthalmic artery originated from the intradural segment (75%), paraclinoid segment (15%), or parasellar segment (10%) of the internal carotid artery. The cross-sectional view of the internal carotid artery through the EEA showed that the ophthalmic artery originated from the middle 1/3 (75%) or medial 1/3 (25%) of the upper surface of the internal carotid artery. On computed tomography angiography, the ophthalmic artery originated from the middle 1/3 (77%) and medial 1/3 (22%) of the upper surface of the internal carotid artery. All ophthalmic arteries were near the level of the distal dural ring (DDR) of the internal carotid artery, that is, within 3 mm above or below the DDR.

**Conclusions:**

The ophthalmic artery usually originates in the middle 1/3 of the upper surface of the intradural segment of the internal carotid artery within 3 mm of the DDR. The ophthalmic artery can be protected to the utmost extent after its origin is identified through an EEA.

## Introduction

Surgery via an endoscopic endonasal approach (EEA) is considered a standard procedure for specific central and paracentral skull base lesions, such as craniopharyngioma and tuberculum sellae meningioma. EEAs have also been used for clipping paraclinoid aneurysms [14]. Early occlusion of the tumor blood supply, proximal internal carotid artery (ICA) control, and decompression of the optic nerve can be achieved through an EEA, unlike through a craniotomy [2]. Accessing the cranial cavity from the nasal cavity has a steep learning curve; however, damage to major nervous and vascular structures within the surgical region, such as the ICA and its branches, venous sinuses, and cranial nerves, must be avoided. Therefore, endoscopic skills and anatomical knowledge are essential for safe and successful surgery [8].

Given the application of various new EEA technologies, such as optic canal decompression [1], the endonasal transoculomotor triangle approach [4], and endonasal anterior clinoidectomy [15], it is of utmost importance to update the anatomy of the origin of the ophthalmic artery (OA) from the perspective of an EEA. There have been many published studies on the anatomy of the origin of the OA from a craniotomy view [3, 6, 7, 11], but that from the ventral view of an EEA has rarely been mentioned [12, 16]. This study aimed to further define the anatomical features of the OA origin from an EEA view. Cadaveric head specimens and computed tomography angiography (CTA) data of normal people were used to (1) understand the position of the OA origin relative to the ICA cross-section from an endoscopic view and (2) clarify the association between the OA origin and the distal dural ring (DDR).

## Materials and methods

### Endoscopic cadaveric dissection

**Fig. 1:**
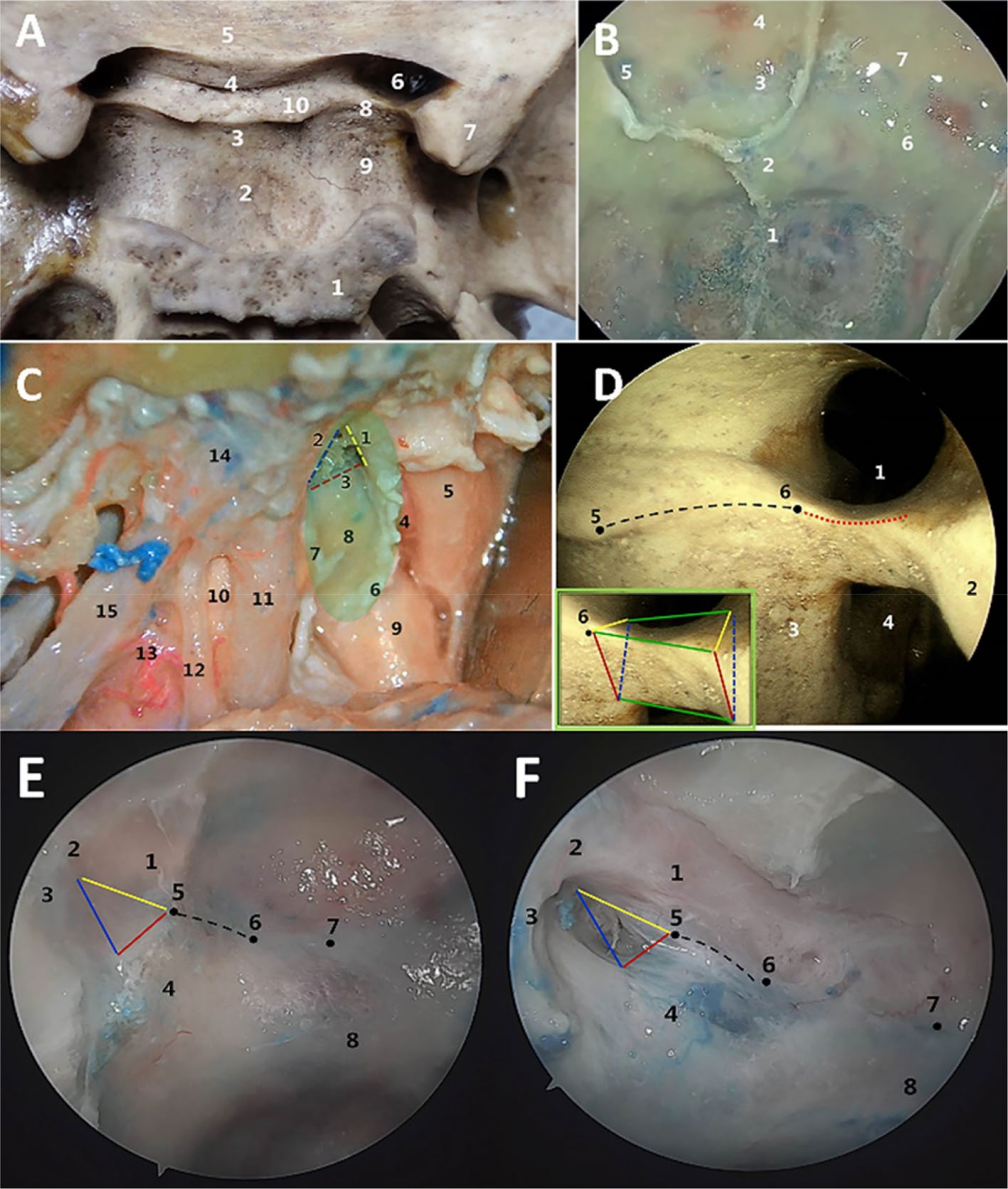
Anatomical study of the sellar region. **A** Bony markers from a dorsal view of the sellar region. 1 = Dorsum sellae, 2 = sellar floor, 3 = tuberculum sellae, 4 = optic groove, 5 = planum sphenoidale, 6 = optic canal, 7 = anterior clinoid process, 8 = inferior optic arch, 9 = carotid groove, 10 = lateral tubercular crest (LTC). **B** Bony markers from a ventral view of the sellar region. 1 = Clival depression, 2 = sellar floor, 3 = tuberculum sellae, 4 = planum sphenoidale, 5 = LOCR, 6 = carotid eminence, 7 = optic nerve eminence. **C** Lateral view of the parasellar region after the anterior clinoid process was epidurally drilled away through a left frontotemporal orbitozygomatic approach. The optic nerve, ICA, and superior orbital fissure are located on the upper, posterior, and lower sides of the optic strut, respectively. The green shadow indicates the range of the drilled anterior clinoid process. 1 = Upper side of the optic strut, 2 = lower side of the optic strut, 3 = posterior side of the optic strut, 4 = OA, 5 = optic nerve, 6 = DDR, 7 = proximal ring, 8 = paraclinoid segment of the ICA, 9 = intradural segment of the ICA, 10 = parasellar segment of the ICA, 11 = oculomotor nerve, 12 = trochlear nerve, 13 = inferolateral trunk of the ICA, 14 = dura mater of the superior orbital fissure, 15 = first branch of the trigeminal nerve. **D** Bony markers of the parasellar region. The optic strut is the posterior root of the lesser wing of the sphenoid bone, an irregular, triangular prismatic bone bridge. The upper surface of the optic strut (two yellow lines and two green lines) forms the base of the optic canal, its lower surface (two blue dashed lines and two green lines) forms the roof of the superior orbital fissure, and its posterior surface (two red lines and two green lines) slopes medially to blend with the carotid groove. 1 = Optic canal, 2 = anterior clinoid process, 3 = carotid groove, 4 = superior orbital fissure, 5 = LTC, 6 = medial point of the optic strut. **E**, **F** Images before and after bone drilling, taken from a ventral view of the parasellar region under a 30° endoscope. The LOCR is a triangular bony depression formed by the ventral surface of the optic strut on the posterior wall of the sphenoid sinus (the red, yellow, and blue indicator lines correspond to the lower, upper, and posterior surfaces, respectively). The LTR is a bony depression located at the most lateral edge of the tuberculum sellae and the medial carotid eminence, corresponding to the LTC in a dorsal view. The LTR is connected to the medial point of the LOCR through the bulged bone arch labeled the DOA (dashed line). 1 = Optic nerve, 2 = orbital apex, 3 = superior orbital fissure, 4 = ICA, 5 = medial point of the LOCR, 6 = LTR, 7 = tuberculum sellae, 8 = sellar floor (colour figure online)

Ten fresh cadaveric heads were subjected to an endoscopic anatomical study through an extended transsphenoidal approach. Standard endonasal instruments (Hotry, NT-1; Xuzhou, China) and rod-lens endoscopes (Bolang, 4 mm, 18 cm; Tianjin, China) were used in cadaveric dissection. Bilateral anterior and posterior ethmoidectomy was conducted. After entering the sphenoid sinus, the skull base was drilled, and the area of bone exposure included the posterior wall of the sphenoid sinus as the anterior boundary, the anterior skull base as the superior boundary, the medial orbital wall as the lateral boundary, and the clivus depression as the inferior boundary. The carotid eminence, optic nerve eminence, lateral opticocarotid recess (LOCR), tuberculum sellae, lateral tubercular recess (LTR), and distal osseous arch (DOA) of the carotid groove were observed (Fig. 1).

The bone on the surface of the ICA was first drilled to produce a thin layer, and then the bone fragment was carefully removed with a disc peeler and Kerrison rongeur for epidural exposure.

Parasellar dural incision: a hook knife was used to first open the dura mater between the ICA and the sellar floor, which corresponded to the medial wall of the cavernous sinus, having the widest relative space and lacking vessels and nerves. Then, the dura mater was separated from the ICA surface with two hands, and the proximal and distal dural rings could be seen to originate from the lower and upper inner edges of the LOCR, respectively. Sellar dural incision: an incision was made above the sellar floor across the superior intercavernous sinus, the dura mater of the tuberculum sellae, and the optic groove, and tissues above the optic nerve sheath were retracted. The diaphragma sellae was cut backward to the infundibulum and outward to the distal dural ring (DDR) to enlarge the area of dural flap exposure. The optic nerve sheath was cut from the inside out, during which the tips of the scissors were kept visible to avoid damage to the OA.

**Fig. 2:**
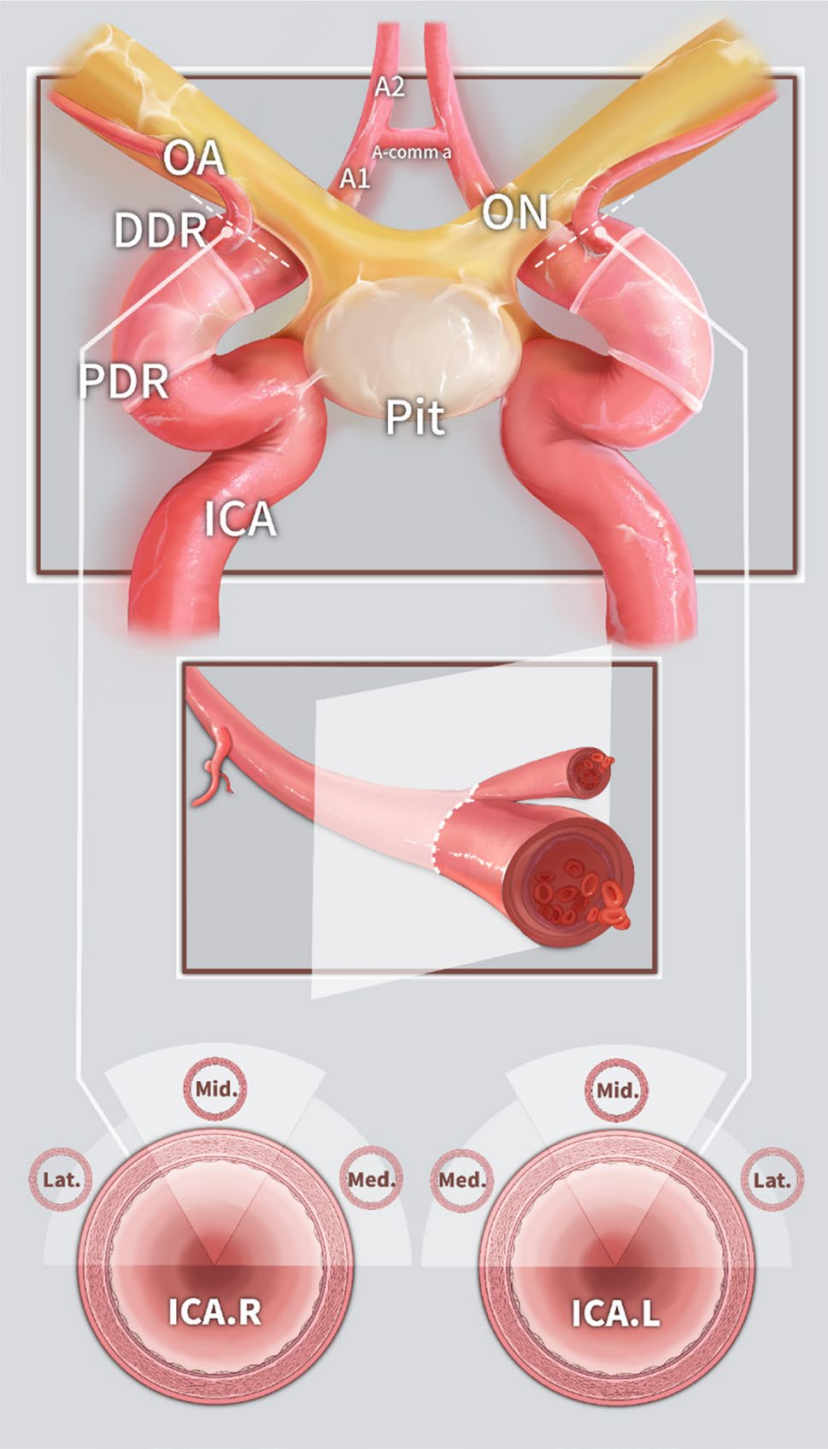
Illustration showing the origin of the OA from the ICA under the EEA view. *A1, A1* segment of the anterior cerebral artery, *ICA* internal carotid artery, *DDR* distal dural ring, *L* left, *Lat* lateral 1/3 of the upper surface, *Med* medial 1/3 of the upper surface, *Mid* middle 1/3 of the upper surface, *OA* ophthalmic artery, *ON* optic nerve, *PDR* proximal dural ring, *Pit* pituitary gland, *R* right

After exposure of the parasellar, paraclinoid and intradural segments of the ICA, (1) the relative position of the OA origin in the cross-sectional view of the ICA was observed (lateral 1/3 of the upper surface, medial 1/3 of the upper surface, middle 1/3 of the upper surface) (Fig. 2), and (2) the distance between the OA origin and the DDR was measured with millimeter tape and a StealthStation™ S8 Surgical Navigation System (Medtronic, Inc.).

Kassam's endoscopic ICA segmentation method was used in this study [9]. The parasellar ICA segment was defined as ranging from the superomedial petrous apex to the proximal dural ring; the paraclinoid ICA segment was defined as ranging from the proximal dural ring to the DDR; and the intradural ICA segment was defined as ranging from the DDR to the ICA bifurcation.

### CTA assessment

After CTA was performed on 50 specimens using a Siemens CT machine (SOMATOM Definition), 3D Slicer software (3D Slicer 5.0) was used to process the image data, and the ventral view of the EEA was reconstructed. Two senior neurosurgeons (Z.C.H. and Z.C.) jointly identified the relative position of the OA origin in the ICA cross-section (lateral 1/3 of the upper surface, medial 1/3 of the upper surface, middle 1/3 of the upper surface).

## Results

**Fig. 3:**
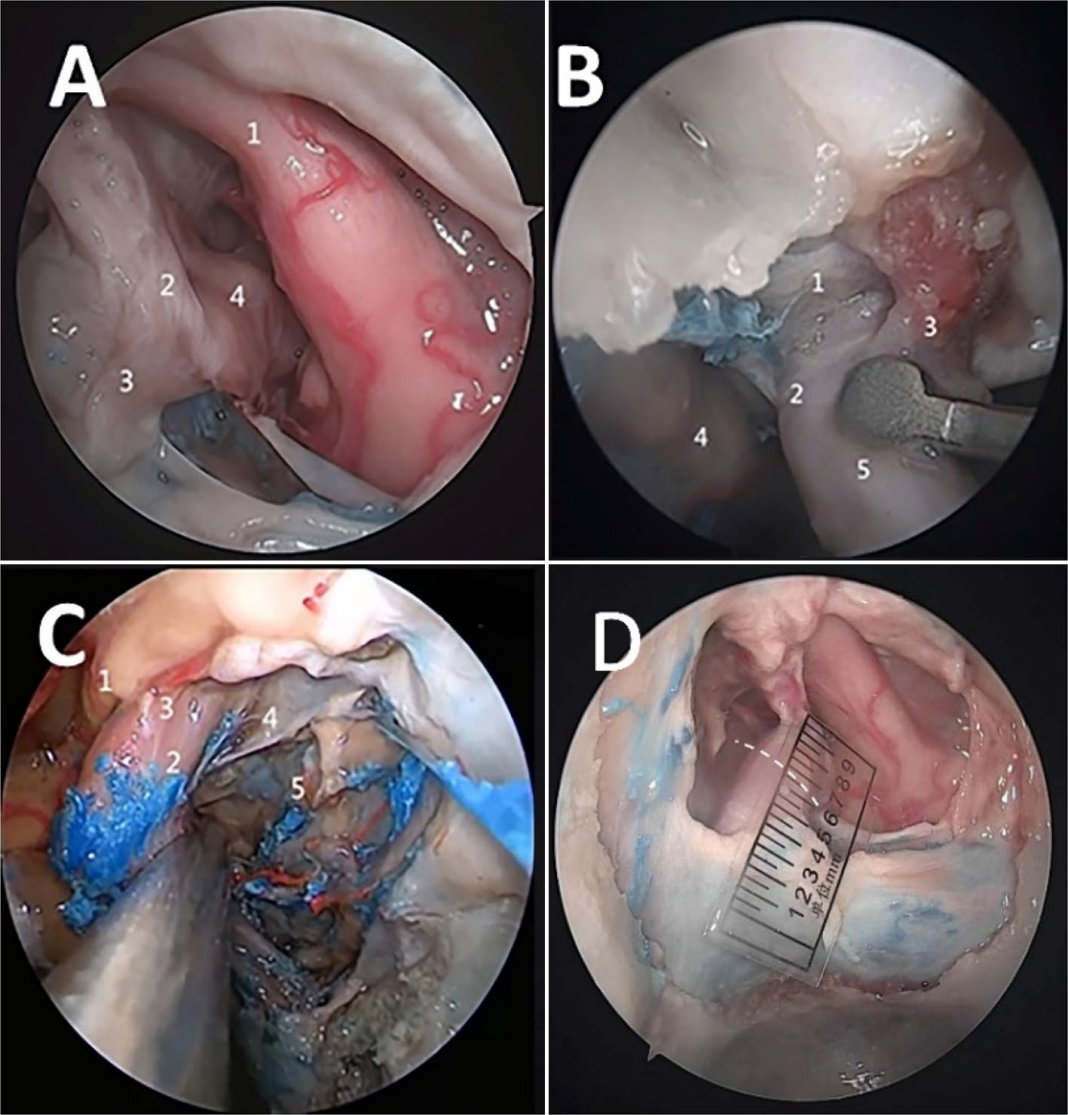
Observation of the OA origin. **A** The right OA arises from the superomedial surface of the intradural segment of the ICA. 1 = Optic nerve, 2 = OA, 3 = DDR, 4 = ICA. **B** The right OA arises from the middle 1/3 of the upper surface of the paraclinoid segment of the ICA. 1 = DDR, 2 = proximal ring, 3 = OA, 4 = oculomotor nerve, 5 = ICA. **C** The left OA arises from the middle 1/3 of the upper surface of the parasellar segment of the ICA. 1 = Optic nerve, 2 = parasellar segment of the ICA, 3 = OA, 4 = carotidoculomotor membrane (proximal ring), 5 = lateral space of the cavernous sinus. **D** The right OA arises from the superomedial surface of the intradural segment of the ICA at a distance of 2 mm from the DDR (white dashed line)

**Fig. 4:**
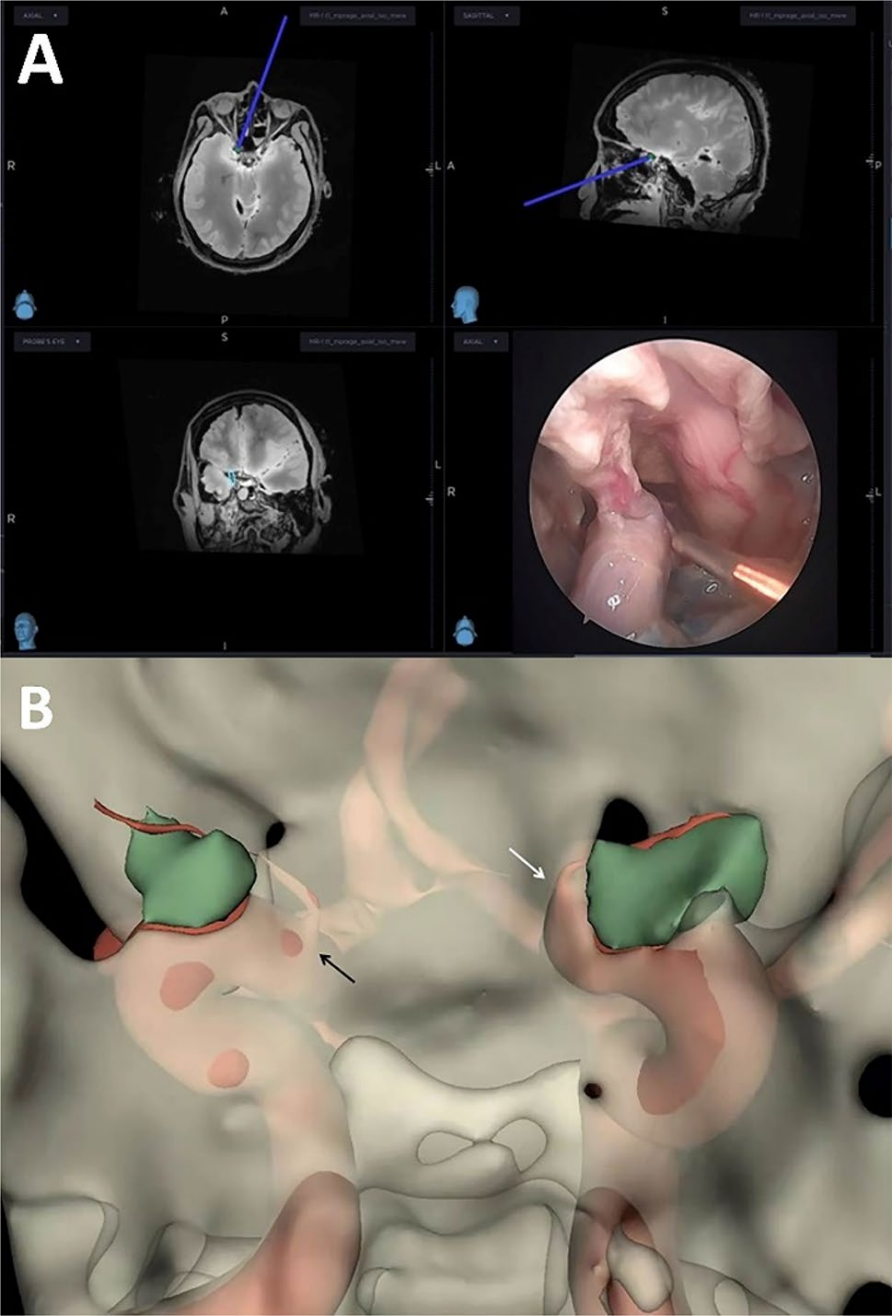
**A** Distance between the OA origin and the DDR measured by electromagnetic navigation. **B** EEA view reconstructed using 3D Slicer software to observe the OA origin; the left side (white arrow) is located on the middle 1/3 of the upper surface of the ICA, the right side (black arrow) is located on the superomedial surface of the ICA, and green indicates the optic strut (colour figure online)

The OA could be observed bilaterally in all specimens, and all sphenoid sinuses showed good pneumatization (Figs. 3 and 4).

### Relative position of the OA origin in the ICA cross-section

In the whole set of cadaveric head specimens, the OA originated from the middle 1/3 of the upper surface of the ICA in 15/20 (75%) sides and the medial 1/3 in 5/20 (25%) sides. As seen on CTA images, the OA originated from the middle, medial, and lateral 1/3 of the upper surface of the ICA in 77/100 (77%), 22/100 (22%), and 1/100 (1%) sides, respectively (Table 1).

**Table 1:** Position of the OA origin in the ICA cross-section

Position of the OA origin in the ICA cross-section	Cadaveric head (%) (*n* = 20)	CTA (%) (*n* = 100)	Total (%) (*n* = 120)
Middle 1/3 of the upper surface	15 (75)	77 (77)	92 (76.7)
Medial 1/3 of the upper surface	5 (25)	22 (22)	27 (22.5)
Lateral 1/3 of the upper surface	0 (0)	1 (1)	1 (0.8)

### Association between the OA origin and the DDR

Among all 20 sides of the cadaveric specimens, the OA origin was located distal to the DDR in 15 sides (75%), with a mean distance between the OA origin and the DDR of 2.2 ± 0.6 mm (0.8–3.0 mm). In the other 5 sides (25%), the OA origin was located proximal to the DDR, including 4 (20%) in the paraclinoid segment and 1 (5%) in the parasellar segment of the ICA, with a mean distance between the OA origin and the DDR of 1.7 ± 0.8 mm (0.6–2.6 mm) and 2.8 mm, respectively. The above findings suggest that the OA may originate near the DDR (within 3 mm), that is, within 3 mm above or below the DOA (Table 2).

**Table 2:** Association between the OA origin and the DDR

Distance from DDR	Intradural Seg. (*n* = 15)	Paraclinoid Seg. (*n* = 4)	Parasellar Seg. (*n* = 1)	Total (*n* = 20)
Mean (mm) ± SD	2.2 ± 0.6	1.7 ± 0.8	2.8	2.1 ± 0.6

*DDR* distal dural ring, *SD* standard deviation, *Seg.* segment

## Discussion

### OA origin in the ICA

From the dorsal perspective in microsurgery, as reported by Rhoton et al., the OA often originates from the site where the ICA exits the cavernous sinus, that is, the proximal end of the intradural segment, after which it runs anteriorly and laterally below the optic nerve to enter the optic canal. In 8% of people, the OA instead originates from the parasellar segment; rarely, it starts from the paraclinoid segment or the middle meningeal artery. It is rarely absent. The OA originates from the middle 1/3 (78%) or the medial 1/3 (22%) of the upper surface of the ICA cross-section and not from the lateral 1/3 of the upper surface [5, 10, 13].

This study found that from the ventral perspective, the OA origin was mostly in the middle 1/3, sometimes in the medial 1/3, and very rarely in the lateral 1/3 of the upper surface of the ICA cross-section. The results obtained from the cadaveric head specimens were similar to those obtained by CTA and similar to the results from the dorsal view. In recent years, some endoscopic studies on the OA origin have been performed and found that all OAs originated in the intradural segment of the ICA [12]. In our study, most OAs originated from the intradural segment, but some also originated proximal to the DDR, which is consistent with the previous results of craniotomy and endoscopic approaches [3, 11, 16]. If this fact is ignored, the OA may be accidentally injured during surgery.

### DDR as a marker of the OA origin

The dural ring is crucial in the endoscopic paracentral skull base approach. The proximal ring is continuous with the carotid-oculomotor membrane, which represents the roof of the cavernous sinus and the beginning of the paraclinoid segment of the ICA. The DDR is closely connected to the adventitia of the ICA and, together with the proximal ring, is continuous posteriorly with the diaphragma sellae. The OA and the superior hypophyseal artery often originate from the ICA near the DDR. The anterior side of the proximal ring and the DDR can be exposed after the bone on the bottom of the carotid groove is drilled away in the medial LOCR. The proximal and distal dural rings must be further opened and fully exposed to treat neoplastic lesions invading the interpeduncular cistern through the oculomotor triangle and paraclinoid aneurysms. As the intradural/epidural interface, the DDR must be opened to enter the subarachnoid space from the paracentral approach.

The diameter of the OA at its origin was 2.25 ± 0.3 mm on the right and 2.16 ± 0.4 mm on the left [3], which is too small to allow accurate localization using current navigation systems; thus, it is necessary to use characteristic markers as references. In some studies, the medial opticocarotid recess (MOCR) has been used as an important marker for locating the OA origin [12]. The MOCR is a teardrop-shaped depression at the junction of the tuberculum sellae, the paraclinoid ICA eminence, the optic canal, and the planum sphenoidale [9]; localizing the MOCR can be complicated, and it cannot be clearly observed at the sellar floor in all cases. As such, the MOCR was not used as a marker in this study. In contrast, the DDR is simple to locate via an EEA, namely, as the dura mater below the DOA, and it objectively exists in all specimens. The OA origin was within 3 mm above or below the DDR, according to the tape measure and electromagnetic navigation.

**Fig. 5:**
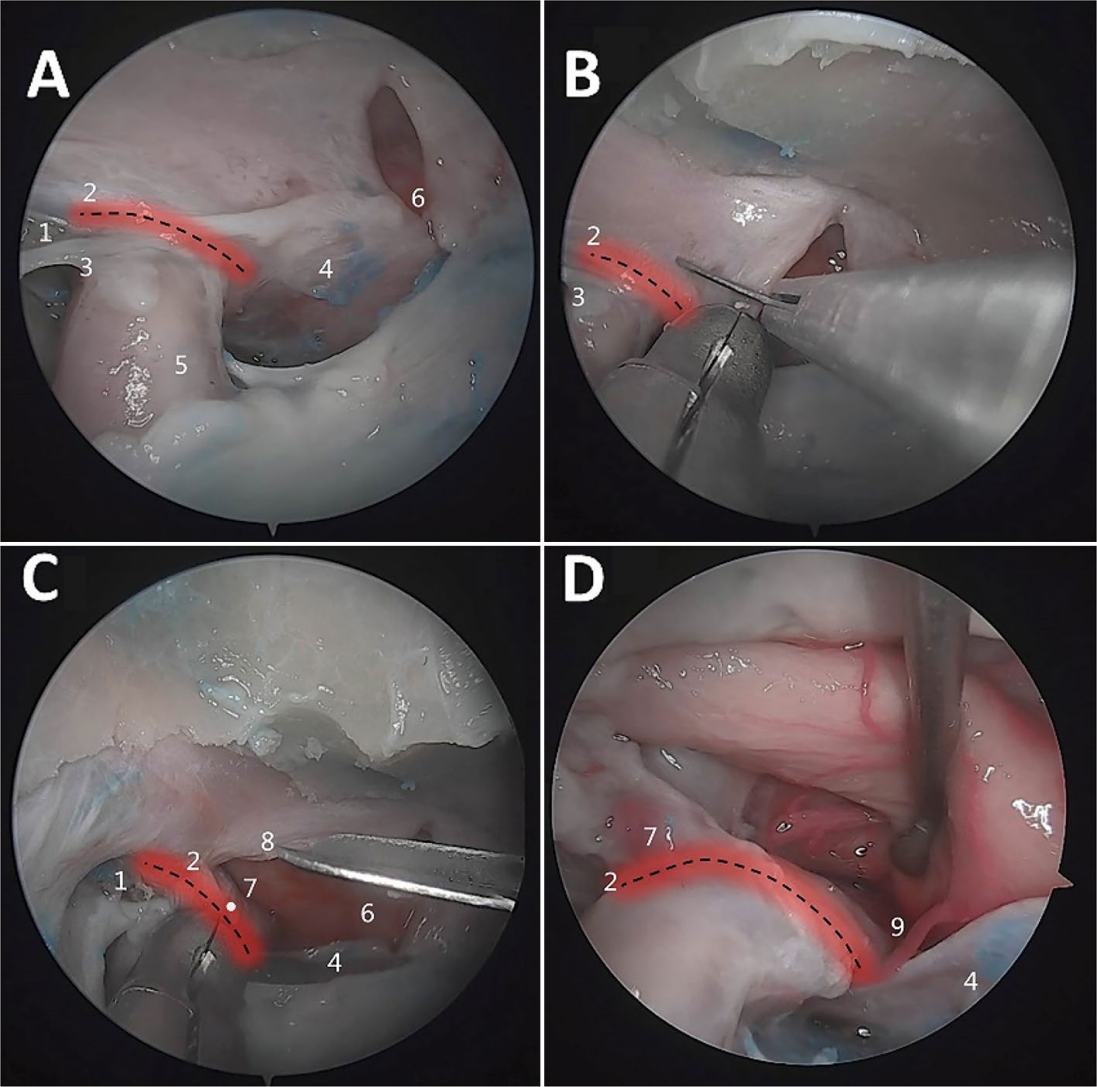
Exposure of the OA. **A** The black dotted line is the projection of the anterior DDR, and the red shadow shows the 3-mm area above and below the DDR, namely, the common origin of the OA. **B** Before cutting the optic sheath, the common origin of the OA should be avoided. **C** After the optic sheath is opened, a dural flap is raised upward to expose the OA origin (white spot). **D** The OA originates from the intradural segment of the ICA as observed in a magnified view. 1 = LOCR, 2 = DDR, 3 = proximal ring, 4 = diaphragma sellae, 5 = ICA, 6 = incision across the superior intercavernous sinus, dura mater of the tuberculum sellae, and optic groove, 7 = OA origin, 8 = incision in the optic sheath, 9 = superior hypophyseal artery (colour figure online)

In line with the experience reported by Abhinav et al. in making incisions in the dura mater of the optic nerve sheath [1], we opened the optic nerve sheath far from the range of 3 mm above and below the DDR, allowing the OA to be well protected (Fig. 5).

## Limitations

We describe the normal OA origin from the view of an EEA, but because of complex space-occupying lesions and trauma, the origins we encountered during surgery were variable; thus, careful preoperative planning and solid anatomical knowledge of the OA origin are needed. Intraoperative fluoroscopy and color Doppler ultrasound guidance can also ensure safe and successful surgery.

## Conclusion

The OA originates most often from the middle 1/3 of the upper surface of the intradural segment of the ICA within 3 mm of the DDR. Through anatomical studies of the OA origin from a ventral view, the risk of accidental injury of the OA in an EEA can be minimized.

## Data Availability

The datasets generated and/or analyzed during the current study are available from the corresponding author on reasonable request.

## Abbreviations

CTA: Computed tomography angiography
DDR: Distal dural ring
DOA: Distal osseous arch
EEA: Endoscopic endonasal approach
ICA: Internal carotid artery
LOCR: Lateral opticocarotid recess
LTR: Lateral tubercular recess
MOCR: Medial opticocarotid recess
OA: Ophthalmic artery
